# The Gene Ontology Annotation (GOA) Project—Application of GO in SWISS-PROT, TrEMBL and InterPro

**DOI:** 10.1002/cfg.235

**Published:** 2003-02

**Authors:** Evelyn Camon, Daniel Barrell, Catherine Brooksbank, Michele Magrane, Rolf Apweiler

**Affiliations:** 1 European Bioinformatics Institute (EBI) Wellcome Trust Genome Campus Hinxton, Cambridge CB10 1SD UK


**Comparative and Functional Genomics**
*Comp Funct Genom* 2003; **4**: 71–74.
Published online in Wiley InterScience (www.interscience.wiley.com). **DOI:** 10.1002/cfg.235

**Conference Review**

### The Gene Ontology Annotation (GOA) project — application of GO in SWISS-PROT, TrEMBL and InterPro

Evelyn Camon*, Daniel Barrell, Catherine Brooksbank, Michele Magrane and Rolf Apweiler
*European Bioinformatics Institute (EBI), Wellcome Trust Genome Campus, Hinxton, Cambridge CB10 1SD, UK*

**Correspondence to:
Evelyn Camon, European Bioinformatics Institute (EBI), Wellcome Trust Genome Campus, Hinxton, Cambridge CB10 1SD, UK.
E-mail:
camon@ebi.ac.uk, goa@ebi.ac.uk*

Received: 15 October 2002
Accepted: 22 November 2002

**Keywords:** ontology; controlled vocabulary; annotation; proteomics

### The challenge

As proteomics research gains momentum, biologists need new ways to access and analyse information on proteins. Many new gene products, from a wide range of species, are being added to the SWISS-PROT Protein Knowledgebase — the world's most highly annotated protein sequence database — and its supplement, TrEMBL [3]. To fully exploit the potential of these data, the SWISS-PROT group at EBI aims to capture all the available biological information related to these sequences and especially components of the human proteome. One important challenge in this endeavour is to make all our databases describe, in a consistent way, what each protein does.

### What is the Gene Ontology Annotation (GOA) project?

GOA is a project run by the European Bioinformatics Institute (EBI) that aims to provide assignments of gene products to the Gene Ontology (GO) resource — a dynamic controlled vocabulary that can be applied to all organisms, even while our understanding of the roles of genes and their products in cells is accumulating and changing [1]. In the GOA project, this vocabulary is being applied to a non-redundant set of proteins described in the EBI's core genome and proteome databases (SWISS-PROT, TrEMBL and Ensembl [8,11] that collectively provide complete proteomes for humans and other organisms.

The GOA project has assigned GO terms, which describe the biological processes, molecular functions and cellular components of a generic cell, to all the complete and incomplete proteomes that exist in SWISS-PROT and TrEMBL, using a combination of electronic mappings and manual curation (see Figure 1). These efforts make the SWISS-PROT group at the EBI one of the largest contributors to the GO consortium annotation effort by providing *over 2.5 million* GO associations across *549917* SWISS-PROT and TrEMBL entries. GOA files are updated on a monthly basis, in accordance with the latest data released by SWISS-PROT, TrEMBL, Ensembl and InterPro (a documentation resource of protein families, domains and sites [4]).

By annotating all characterized proteins with GO terms and facilitating the transfer of this

Copyright © 2003 John Wiley & Sons, Ltd.

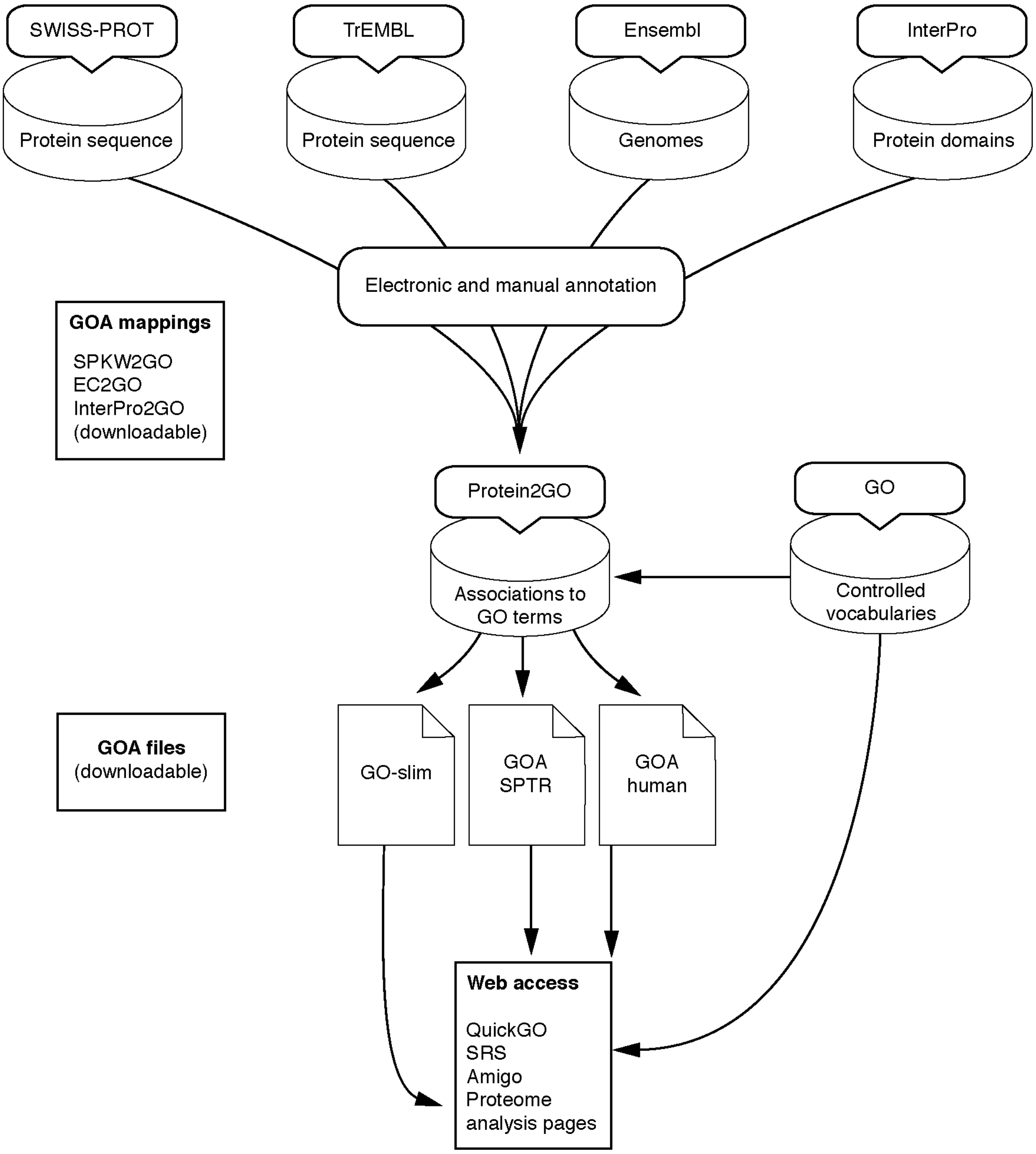

**Figure 1.** GOA dataflow knowledge to similar uncharacterized proteins, we hope to contribute to a better understanding of all proteomes.

### How is GO annotated at the EBI?

The large-scale assignment of GO terms to SWISS-PROT and TrEMBL entries involves electronic techniques. We use existing information within database entries, including keywords, enzyme commission (EC) numbers and cross-references to InterPro [4], which are manually mapped to GO. Electronically combining these mappings with a table of matching SWISS-PROT and TrEMBL entries generates a table of associations. For each GOA association, an evidence code, which

Copyright © 2003 John Wiley & Sons, Ltd. 

summarizes how the association is made, is provided. Associations that are made electronically are labelled as 'inferred from electronic annotation' (IEA).

Manual assignment of GO terms by SWISS-PROT curators uses published literature and provides more reliable GO annotation than electronic annotation. As entries are manually curated, the evidence code 'IEA' is replaced with a code that summarizes the experimental evidence for the association. For further information on evidence codes, please see the GO annotation guide on the GO Home Page [9].

### Retrieving data from GOA

There are various ways of accessing and searching GOA project data, including several web-based browsers (Table 1). The GOA files and mappings can also be downloaded.

### What can I do with GOA?

The success of the Gene Ontology Consortium can already be measured by the number of world-leading academic and commercial databases that use it to annotate and exchange biological knowledge in a consistent manner (see GO Home Page [9]). The GOA Project at EBI has made an enormous contribution to this global effort. We hope that our annotation will contribute to the discovery of the function of new sequences by helping scientists to determine more quickly functional similarities among proteins. Using GOA, you can:

- Find human- and computer-readable functional information on the proteomes of almost 50 000 species.
- Download non-redundant proteome sets for humans and for all the gene products in SWISS-PROT/TrEMBL/InterPro/Ensembl — to incorporate them into your own databases or to validate automated ways of deriving information about gene function.
- Navigate proteomic data repositories in a biologically intuitive and scientifically accurate manner. For example, you can ask SRS to find you all the proteins involved in apoptosis but not involved in the TRAIL pathway, or to find

**Table 1.** Resources for accessing and searching GOA project data

| Resource | Description |
|---|---|
| Web-based tools | |
| QuickGO | A fast web-based browser with access to core GO data and up-to-date electronic and manual EBI GO annotations [15] |
| SRS | Use the EBI's Sequence Retrieval System to search the GOA database or the GO consortium repository [16] |
| Proteome Analysis Pages | GO annotations have been produced for classification of proteins belonging to each complete proteome. A slimmed-down version of GO (GO-slim), representing high-level GO terms, provides an overview of each proteome [14] |
| InterPro | GO annotations made by InterPro are visible directly in InterPro entries [13] |
| AmiGO | This GO Consortium browser provides access to core GO data and released GOA data [1] |
| Downloads | |
| GOA Association File | This is a tab-delimited file of associations between gene products and GO terms. Two GOA association files are currently produced: the human GOA file contains GO annotations for all proteins in the non-redundant human proteome set; the SPTR GOA file contains GO annotations for all proteins in SWISS-PROT and TrEMBL. Both files can be accessed from the EBI ftp server [6] |
| GOA Xref File | This is a file of cross-references that displays the relationship between the entries in the GOA data set with other databases using International Protein Index (IPI) [12], as well as the nucleotide sequence databases, HUGO, LocusLink and Refseq |
| InterPro2GO Mapping | This is a manual mapping of InterPro domains to GO terms maintained at the EBI [13] |
| SPKW2GO Mapping | This is a manual mapping of SWISS-PROT keywords to GO terms maintained at the EBI [17] |
| EC2GO Mapping | This is a manual mapping of Enzyme Commission Numbers to GO terms maintained by the GO Consortium [7] |

Copyright © 2003 John Wiley & Sons, Ltd.

you all the cytokines that inhibit apoptosis by interacting with the caspase pathway.
- Find all the proteins involved in a biological process, or that form a particular function, e.g. using GO-slim — our summary of GO annotation — you can find all the proteins involved in neurotransmission, or all the kinases, in a dataset.
- Map GO terms to your own datasets; e.g. our annotation of InterPro terms (InterPro2GO [13]) can be used to transfer functional information to mass spectrometry data or microarray data.
- Support development of GO by requesting new terms to enhance the ontologies and improve their specificity.

For more information about applications of GO annotation, please see the studies cited at **www.geneontology.org/doc/GO.biblio.html**

### Can I get GO annotation for nucleotide sequences?

To support the mapping of biological knowledge, and especially to facilitate the interpretation of genomic data, we plan to make GOA accessible directly in the EMBL-Bank flat files, which contain the nucleotide sequences of the international collaboration EMBL-Bank/GenBank/DDBJ.

### The future of GOA

At the outset of the GOA project, only SWISS-PROT and InterPro curators at the EBI participated. With the recent announcement of the UniProt Consortium [4], the existing collaboration between SWISS-PROT at the EBI and Swiss Institute of Bioinformatics (SIB), Geneva, has been extended to encompass the Protein Information Resource (PIR), USA. With more curators manually annotating GO terms, more of the knowledge contained in SWISS-PROT and TrEMBL databases will become computationally accessible. Currently GOA covers 64% of the SWISS-PROT and TrEMBL data; by 2004 we aim to have assigned GO terms to over 70% of all SWISS-PROT and TrEMBL proteins. Plans to integrate manual GO assignments made by other GO Consortium groups, e.g. FlyBase, will further enhance the GOA dataset. We also plan improvements to our GO querying interfaces, SRS and QuickGO.

### Contributing to the GOA project

The success and accuracy of GOA rely on frequent electronic and manual checking. If you find anything in GOA that needs correcting or updating, please let us know by sending an e-mail to **goa@ebi.ac.uk**. Please provide the accession number of the entry you are enquiring about, along with the source and date of annotation retrieval. If your group wishes to share manual GO annotation, then please contact GOA and/or the GO consortium [9] for further information on the agreed format for data exchange.

### Acknowledgements

GOA is supported by the European Commission and the US National Institutes of Health.

### References

1. AmiGO Browser: **http://www.godatabase.org/cgi-bin/go.cgi**
2. Apweiler R, Biswas M, Fleischmann W, *et al*, 2001. Proteome Analysis Database: online application of InterPro and CluSTr for the functional classification of proteins in whole genomes. *Nucleic Acids Res* **29**(1): 44–48.
3. Bairoch A, Apweiler R. 2000. The SWISS-PROT protein sequence database and its supplement TrEMBL in 2000. *Nucleic Acids Res* **28**(1): 45–48.
4. Biswas M, O'Rourke JF, Camon E, *et al*. 2002. Applications of InterPro in protein annotation and genome analysis. *Brief Bioinform* **3**(3): 285–295.
5. Butler D. 2002. NIH pledges cash for global protein database. *Nature* **419**: 101.
6. EBI FTP Server: **ftp://ftp.ebi.ac.uk/pub/databases/GO/goa**
7. EC2GO Mapping: **http://www.geneontology.org/external2go/ec2go**
8. Ensembl: **http://www.ensembl.org/Homo_sapiens/**
9. Gene Ontology Home Page: **http://www.geneontology.org/**
10. Gene Ontology Annotation Home Page: **http://www.ebi.ac.uk/GOA**
11. Hubbard T, Barker D, Birney E, *et al*, 2002. The Ensembl genome database project. *Nucleic Acids Res* **30**(1): 38–41.
12. International Protein Index: **http://www.ebi.ac.uk/IPI/IPIhelp.html**
13. InterPro2GO Mapping: **http://www.geneontology.org/external2go/interpro2go**
14. Proteome Analysis Pages: **http://www.ebi.ac.uk/proteome**
15. QuickGO Browser: **http://www.ebi.ac.uk/ego/QuickGO**
16. Sequence Retrieval System (SRS): **http://srs.ebi.ac.uk/**
17. SWKW2GO Mapping: **http://www.geneontology.org/external2go/spkw2go**
18. The Gene Ontology Consortium. 2001. Creating the gene ontology resource: design and implementation. *Genome Res* **11**: 1425–1433.

Copyright © 2003 John Wiley & Sons, Ltd. 
